# Artificial Intelligence and Patient Autonomy in Obesity Treatment Decisions: An Empirical Study of the Challenges

**DOI:** 10.7759/cureus.49725

**Published:** 2023-11-30

**Authors:** Turki Alanzi, Ahlam Alhajri, Sara Almulhim, Sara Alharbi, Samya Alfaifi, Eslam Almarhoun, Raghad Mulla, Zainab O Alasafra, Zainab Alalwan, Fatima Alnasser, Fatima Almukhtar, Fatemah Al Ghadeer, Sara Amro, Ibrahim Alodhayb, Nouf Alanzi

**Affiliations:** 1 Department of Health Information Management and Technology, College of Public Health, Imam Abdulrahman Bin Faisal University, Dammam, SAU; 2 College of Agricultural and Food Sciences, King Faisal University, Al Hofuf, SAU; 3 College of Medicine, King Faisal University, Al Hofuf, SAU; 4 College of Applied Medical Sciences, Umm Al-Qura University, Makkah, SAU; 5 College of Pharmacy, Umm Al-Qura University, Makkah, SAU; 6 Family Medicine, South Khobar Primary Healthcare Center, Khobar, SAU; 7 ‏College of Medicine, King Abdulaziz University, Jeddah, SAU; 8 Family Medicine, Doha Primary Health Center, Dammam, SAU; 9 College of Medicine, Imam Abdulrahman Bin Faisal University, Dammam, SAU; 10 Medicine and Surgery, King Fahad University Hospital, Khobar, SAU; 11 Department of Pharmacy, Al Moosa Specialist Hospital, Al Mubarraz, SAU; 12 Family Medicine, King Fahad Armed Forces Hospital, Jeddah, SAU; 13 College of Agriculture and Veterinary Medicine, Qassim University, Buraydah, SAU; 14 Department of Clinical Laboratory Sciences, College of Applied Medical Sciences, Jouf university, Sakakah, SAU

**Keywords:** treatment, decision-making, patients, trust, autonomy, artificial intelligence, obesity

## Abstract

Background

This study aims to explore the factors associated with artificial intelligence (AI) and patient autonomy in obesity treatment decision-making.

Methodology

A cross-sectional, online, descriptive survey design was adopted in this study. The survey instrument incorporated the Ideal Patient Autonomy Scale (IPAS) and other factors affecting patient autonomy in the AI-patient relationship. The study participants included 74 physicians, 55 dieticians, and 273 obese patients.

Results

Different views were expressed in the scales AI knows the best (μ = 2.95-3.15) and the patient should decide (μ = 2.95-3.16). Ethical concerns (μ = 3.24) and perceived privacy risks (μ = 3.58) were identified as having a more negative influence on patient autonomy compared to personal innovativeness (μ = 2.41) and trust (μ = 2.85). Physicians and dieticians expressed significantly higher trust in AI compared to patients (p < 0.05).

Conclusions

Patient autonomy in the AI-patient relationship is significantly affected by privacy, trust, and ethical issues. As trust is a multifaceted factor and AI is a novel technology in healthcare, it is essential to fully explore the various factors influencing trust and patient autonomy.

## Introduction

The integration of artificial intelligence (AI) into healthcare has ushered in a new era of medical innovation, promising to enhance diagnostic accuracy, optimize treatment strategies, and improve patient outcomes. Within this rapidly evolving landscape, the application of AI in the domain of obesity treatment decision-making holds immense potential [1,2]. Obesity, a complex and multifaceted health condition, affects millions of individuals worldwide, placing them at risk for a myriad of associated health issues, including diabetes, cardiovascular diseases, and certain cancers [3]. The need for effective, personalized treatment strategies is paramount, and AI has emerged as a powerful ally in this process [4]. Patient autonomy (the right of patients to make decisions about their medical care without influence from their healthcare provider or system), a cornerstone of medical ethics, underscores the importance of a patient’s right to make informed decisions about their own healthcare [5]. The introduction of AI in the context of obesity treatment decision-making presents a delicate balancing act, where the benefits of AI must harmonize with preserving patients’ autonomy and ensuring that their values and preferences are respected [6].

Obesity management necessitates a comprehensive approach that considers a patient’s unique physiological and psychological factors, lifestyle, and social determinants of health [7]. Obesity treatment is intricately linked to diet and nutrition, forming the cornerstone of effective management. This multifaceted approach focuses on regulating caloric intake, promoting a balanced diet rich in nutrient-dense foods, and adopting healthy eating habits [8,9]. Reducing portion sizes and adhering to regular eating schedules can be instrumental in curbing calorie consumption, while mindful eating fosters a deeper connection to hunger and fullness cues. Cutting back on sugary and processed foods, alongside maintaining proper hydration, plays a pivotal role in weight control [10]. Ultimately, seeking guidance from healthcare professionals, such as registered dietitians or nutritionists, can provide tailored nutrition plans and strategies to address the complexities of obesity while ensuring long-term success [11]. For instance, an AI-integrated diabetes treatment approach using the Internet of Things, where processes such as continuous glucose monitoring are automated and the data is analyzed using AI technologies aiding nutritionists and physicians in preparing treatment plans, has resulted in significant positive outcomes such as diabetes reversal [12,13]. From these studies [12,13], it is evident that AI technologies enable individuals with diabetes to access a wealth of real-time data and personalized insights, allowing them to make informed decisions about their treatment and lifestyle choices. Through data analysis, AI can create highly individualized treatment plans, respecting the unique needs and preferences of each patient. This personalized approach enhances patient autonomy by giving them more control over their diabetes management. It also promotes a collaborative relationship between patients and healthcare providers, as individuals can actively participate in the decision-making process while relying on the support of AI tools for better self-management and adherence to treatment plans.

However, as AI becomes increasingly ingrained in the decision-making processes surrounding disease treatment, it raises critical questions concerning patient autonomy and ethical considerations [14]. It is important to strike a balance, ensuring that patients fully understand and are comfortable with the technology, maintaining transparency, and safeguarding their right to accept or decline AI-based recommendations. AI technologies offer the promise of sifting through vast amounts of data to generate personalized treatment plans, making healthcare more precise and effective [15]. Yet, as AI systems become involved in the decision-making process, concerns emerge regarding the extent to which patients’ voices are heard and their choices respected. Healthcare providers, including physicians, dietitians, and therapists, play a pivotal role in navigating the intersection of AI and obesity treatment, ensuring that technological advancements enhance rather than impede the doctor-patient relationship [16]. Patients, conversely, must contend with the complexities of AI-generated recommendations and strike a balance between trusting these technologies and exercising their own judgment. The analysis of the increasing autonomy in healthcare has primarily focused on its role in empowering patients. However, research in this area has indicated that this concept also brings about novel mechanisms of social control over patients’ lives. Additionally, it presents challenges for patients who are unable to conform to the professionals’ normative expectations regarding self-management and self-care [17]. It was also observed that in relation to autonomy, beneficence, non-maleficence, and justice, the current provision of bariatric surgical care fell short of meeting internationally recognized medical ethical standards [18]. While these findings are in the context of the doctor-patient relationship, there is a dearth of research in relation to patient autonomy in the doctor-AI-patient relationship. Therefore, this study aims to investigate the challenges associated with AI use and patient autonomy in obesity treatment decision-making.

This empirical study delves into the challenges encountered in the utilization of AI for obesity treatment decisions, with a particular focus on how this integration may impact patient autonomy. The perspectives and experiences of both healthcare professionals and patients are instrumental in understanding the multifaceted nature of these challenges. This study endeavors to explore the multifaceted challenges faced by both healthcare providers and patients in the context of AI-assisted obesity treatment decision-making. By shedding light on these challenges, we aspire to identify potential solutions, ethical considerations, and strategies that enable the harmonious coexistence of AI and patient autonomy, ultimately working toward more effective and patient-centered obesity management.

## Materials and methods

Recruitment and sampling

As the study focused on obesity, the participants included physicians and patients from public hospitals and primary care centers. As participants were purposively recruited from the selected hospitals, convenience and purposive sampling techniques were adopted [19]. The inclusion criteria included physicians who were using or aware of AI-powered virtual assistants for not less than three months in clinical decision-making and treating obesity. Only obese patients who were aware of or were using AI-powered virtual assistants for self-help and disease management were included in the study.

Instruments

The survey questionnaire was divided into two sections. The first section focused on collecting demographic information related to age, gender, education, role, and experience with AI-assisted technologies. The second section focused on collecting data on patient autonomy and AI technology-related factors. AI knows best (five items), patient should decide (four items), right to non-participation (three items), and obligatory risk information (two items) were adopted from the pre-validated Ideal Patient Autonomy Scale (IPAS) [20]. In addition, behavioral intention (BI) (three items) was adapted from Joshi [21]; three factors including perceived privacy risks (PPR) (four items), trust (four items), and personal innovativeness (PI) (four items) were adopted from García de Blanes Sebastián et al. [22]; and ethical concerns (ECs) (five items) were adapted from Kooli et al. [23]. Furthermore, recommendations for using AI in obesity treatment decision-making (five items) and benefits and challenges (six items) related to AI in obesity treatment were developed by the authors. The questionnaire was designed using Google Forms by creating a link to access the survey. A pilot study was conducted among 14 physicians and the data were analyzed. Cronbach’s alpha was calculated for all items and was observed to be greater than 0.7, indicating good internal consistency [24].

Ethical considerations

All participants were fully informed about the study through an information sheet attached to the invitation email. Informed consent was obtained from all participants using a check button before starting the survey. The participation was voluntary and the participants were assured of their anonymity and their rights with respect to the data. Ethical approval was received from the Ethics Committee at Imam Abdulrahman Bin Faisal University.

Data collection

A participant information sheet was attached along with the invitation email (containing the survey link), explaining the rights of the participants, and forwarded to all the physicians, dieticians, and patients who agreed to participate in the survey. A total of 283 patients and 89 physicians participated in the survey.

Data analysis

To attain the objectives of the research, the researcher utilized SPSS version 24 (IBM Corp., Armonk, NY, USA) for analyzing the data. Descriptive statistics was used to characterize the participants’ demographic data. In addition, the two-sample t-test with unequal variances was used for analyzing the data. Furthermore, Pearson’s correlation coefficients were used to compare the relationship between various factors.

## Results

A total of 402 individuals participated in the study, of whom 74 were physicians, 55 were dieticians, and 273 were patients (suffering from obesity). The majority of the participants were males, representing 64.4% of the total participants. Detailed demographic information of the participants is presented in Table 1.

**Table 1:** Participants’ demographics.

Variable		N	Relative frequency
Age (in years)	18–30	186	46.3%
	31–40	82	20.4%
	41–50	84	20.9%
	51–60	50	12.4%
Gender	Male	259	64.4%
	Female	143	35.6%
Education	Primary/Secondary education	62	15.4%
	Diploma	57	14.2%
	Bachelor’s degree	241	60.0%
	Master’s degree	37	9.2%
	Other	5	1.2%
Role	Physicians	74	18.4%
	Dieticians	55	13.7%
	Patients	273	67.9%

**Figure 1:**
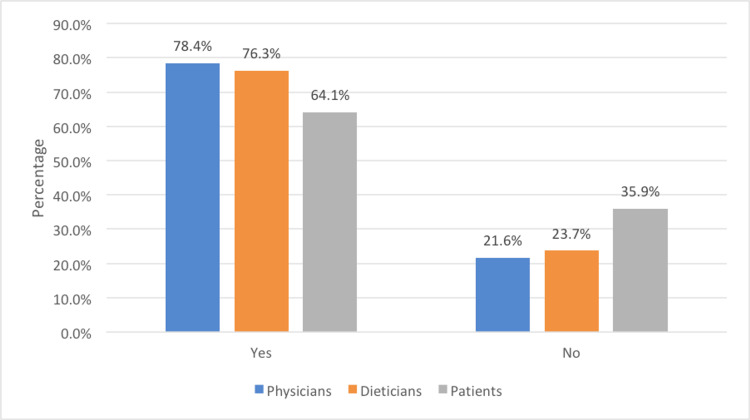
Usage of artificial intelligence (AI)-enabled technologies by the participants.

Among the total participants, 76.3% had used AI-enabled technologies for obesity treatment and care. The usage according to participants’ roles is presented in Figure 1.

The data from Table 2, which presents the mean ratings of the IPAS sub-scales, revealed a nuanced perspective on the role of AI in healthcare decision-making. The AI knows the best (AKB) sub-scale revealed a general inclination, albeit subtle, toward a preference for AI-driven decision-making, where both patients and professionals rated statements indicating trust in AI’s decision-making slightly higher than patient-centered decisions. However, there is a noticeable nuance in the responses, with patients slightly more inclined toward autonomous decision-making than professionals. The patient should decide (PSD) sub-scale showed a higher preference among patients for their own decision-making compared to the perspectives of professionals. On the other hand, the right to non-participation (nonP) sub-scale indicated a balanced perspective between patients and professionals, suggesting a consensus on respecting patients’ rights to involvement or non-involvement in treatment decisions. Lastly, the obligatory risk information (ORI) sub-scale indicates a strong agreement across both groups, emphasizing the necessity of informing patients about treatment risks, with patients rating this slightly higher than professionals. Overall, while there is a trend suggesting a degree of trust in AI’s decision-making, the data indicated a nuanced balance between patient autonomy and AI involvement, highlighting the importance of informed consent and patient-centered care in healthcare decision-making processes.

**Table 2:** Mean ratings of the Ideal Patient Autonomy Scale (IPAS).

Sub-scale	Items	Mean (μ)	
		Patients	Physicians and dieticians
AI knows the best (AKB)	If the AI assistant and patient cannot agree on which treatment is best, then the AI assistant should make the treatment decision	3.15	3.14
	It is better that the AI assistant rather than the patient decides which is the best treatment	3.01	3.05
	During the conversation, the patient must submit himself/herself with confidence to the expertise of the AI assistant	3.13	2.95
	The AI assistant can presume that the patient knows that people can die during serious operations/mistreatment	3.05	2.96
	The patient should, without much information about the risk involved, confidently undergo an operation/follow treatment	2.94	2.98
Patient should decide (PSD)	The patient himself/herself must choose between the various treatments	3.18	2.95
	If a patient chooses a treatment with more health risks, the AI assistant should respect this treatment decision	3.02	2.81
	It goes too far when the AI assistant decides which treatment is best for the patient	3.18	3.12
	As it concerns the body and life of the patient, the patient should decide	3.03	2.93
Right to non-participation (nonP)	If the patient does not want to receive information about risks, the AI assistant should respect this	3.06	3.02
	Patients who become afraid when thinking about the treatment decision should be left in peace by AI	3.16	3.01
	Patients should have the right not to be involved in the decision regarding the treatment	3.18	3.19
Obligatory risk information (ORI)	The patient must be informed about all the risks involved in an operation/treatment	3.31	3.18
	Before a patient consents to a treatment, he/she should receive all information about the risks involved	3.24	3.13

The correlation matrix of the IPAS (Table 3) sub-scales revealed interesting insights into the relationships between these different facets of patient autonomy and the perceived role of AI in healthcare decision-making. First, there was a very weak negative correlation of -0.047 between AKB and PSD, indicating a slight tendency for individuals who trust AI more to be less inclined toward patient autonomy and vice versa. Similarly, a very weak positive correlation of 0.072 existed between AKB and nonP, suggesting that those who favored AI involvement may also support a patient’s right to abstain from decision-making. Meanwhile, a slightly stronger positive correlation of 0.146 between AKB and ORI implied that individuals more trusting of AI also emphasize the importance of providing comprehensive risk information to patients. Overall, these correlations indicate subtle relationships, with no strong connections between these different aspects of patient autonomy and AI involvement.

**Table 3:** Correlation matrix of the Ideal Patient Autonomy Scale (IPAS) sub-scales (Pearson’s r). AKB: AI knows the best; PSD: Patient should decide; nonP: Right to non-participation; ORI: Obligatory risk information

	AKB	PSD	nonP	ORI
AKB	1			
PSD	-0.047	1		
nonP	0.072	-0.010	1	
ORI	0.146	-0.050	0.076	1

**Figure 2:**
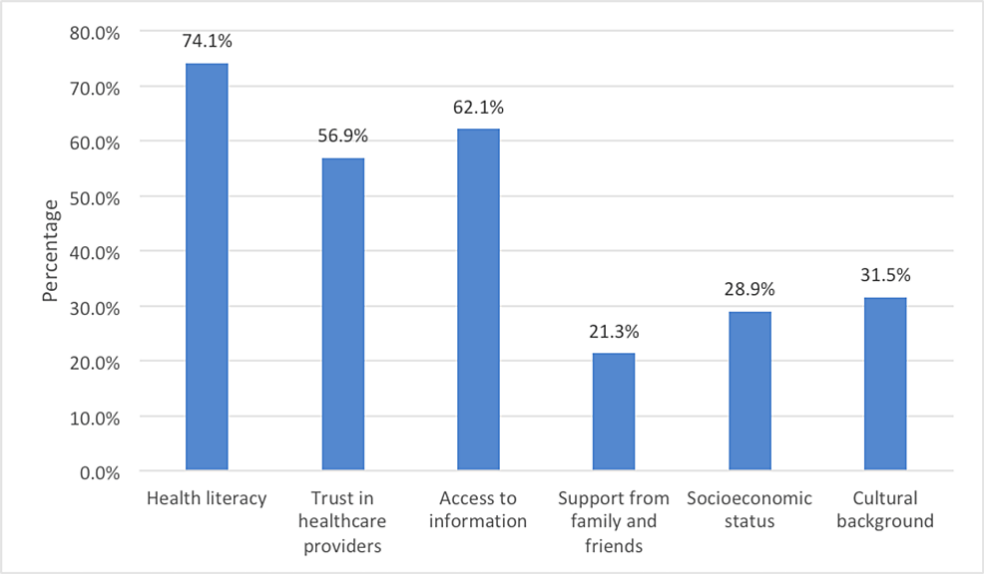
Key factors influencing patient autonomy.

In relation to patient autonomy in obesity treatment and decision-making, 21.5% of the total participants considered it to be extremely important, followed by 39.8% stating it as very important, and 12.3% as important. About 21.6% stated it as somewhat important, and 4.8% stated it as not important. Figure 2 highlights several key factors influencing patient autonomy in healthcare decisions. Health literacy, with a prevalence of 74.1%, emerged as a paramount factor, emphasizing the importance of well-informed patients in making autonomous choices. Trust in healthcare providers at 56.9% underscored the critical role of a patient-provider relationship built on confidence and communication. Access to information, at 62.1%, was pivotal for informed decision-making, emphasizing the need for accessible, reliable healthcare resources. Support from family and friends, at 21.3%, acknowledged the varying influence of personal networks on patient autonomy. Socioeconomic status and cultural background, though with lower prevalence at 28.9% and 31.5%, respectively, still played essential roles, affecting access to care and shaping cultural values that impact autonomy. These factors collectively illustrate the intricate web of influences on patient autonomy, underscoring the need for tailored approaches in healthcare decision-making.

**Figure 3:**
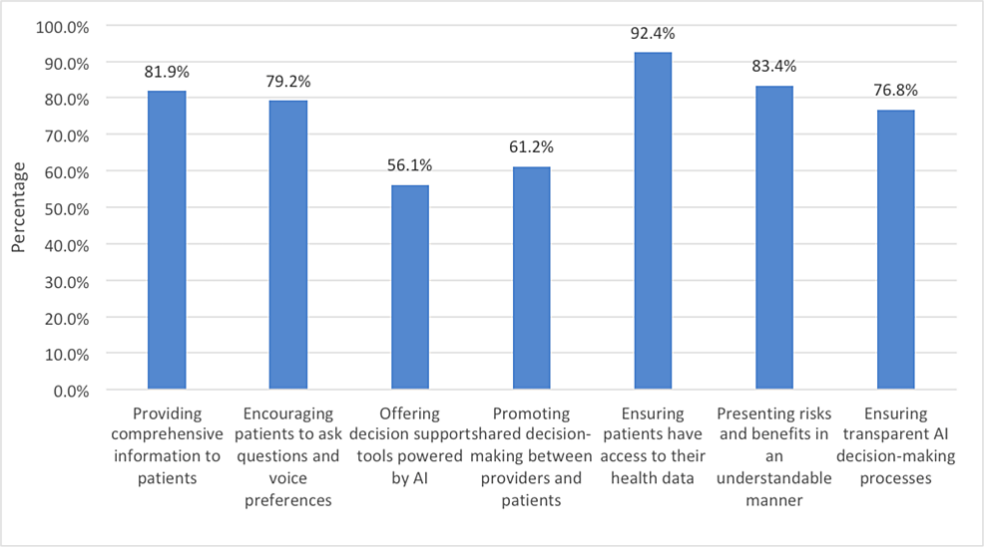
Image illustrating how artificial intelligence (AI) tools can enhance patient autonomy.

Figure 3 underscores the substantial potential of AI tools to enhance patient autonomy in healthcare decision-making. Notably, providing comprehensive information to patients (81.9%) and ensuring access to their health data (92.4%) emerged as powerful means to empower patients with knowledge and control over their healthcare choices. Encouraging patients to ask questions and voice their preferences (79.2%) fosters active involvement while presenting risks and benefits in an understandable manner (83.4%) aids informed decisions. Furthermore, promoting transparent AI decision-making processes (76.8%) and shared decision-making between providers and patients (61.2%) signifies the importance of AI in facilitating collaborative and patient-centered care. Although the utilization of decision support tools powered by AI (56.1%) showed a slightly lower prevalence, it still contributes positively to patient autonomy by offering guidance. Overall, these statistics emphasize the transformative potential of AI tools in promoting patient autonomy and informed decision-making in healthcare.

Among the various factors influencing patients’ autonomy while using AI in decision-making, ethical concerns (EC) (μ = 3.24) and perceived privacy risks (PR) (μ = 3.58) were identified as having a more negative influence on patient autonomy compared to personal innovativeness (PI) (μ = 2.41) and trust (μ = 2.85).

The data in Table 4 reveals participants’ perceptions regarding factors related to AI influencing patients’ autonomy, with a focus on age and role distinctions. In terms of BI, age does not significantly impact perceptions, as both age groups below and above 40 showed similar levels of belief in AI’s influence. Likewise, the role distinctions between physicians/dieticians and patients do not result in significant differences in their beliefs about AI. However, trust in AI displays an interesting trend. While age groups show comparable levels of trust, physicians and dieticians express significantly higher trust in AI compared to patients (p < 0.05), indicating that healthcare professionals may have more confidence in AI. PPR and EC do not exhibit significant differences based on age or role. Perceived impact also showed no substantial variations, highlighting a consistent perception of AI’s influence across ages and roles.

**Table 4:** Differences in participants’ perceptions related to factors related to artificial intelligence (AI) influencing patients’ autonomy. *: statistically significant difference; df: degrees of freedom; SD: standard deviation; BI: behavioral intention; PPR: perceived privacy risk; EC: ethical concern; PI: personal innovativeness

Factors	Age	N	Mean	SD	df	t-value	P-value
BI	≤40 years	268	2.98	1.15	253	0.4213	0.3369
	>40 years	134	3.03	1.29			
	Physician and dietician	129	3.05	1.28	241	0.6831	0.2475
	Patients	273	2.97	1.16			
Trust	≤40 years	268	2.86	1.06	248	0.0891	0.4645
	>40 years	134	2.85	1.24			
	Physician and dietician	129	3.01	1.04	262	2.0757	0.0194
	Patients	273	2.78	1.14			
PPR	≤40 years	268	3.58	0.48	254	0.1705	0.4323
	>40 years	134	3.57	0.54			
	Physician and dietician	129	3.6	0.48	258	0.3799	0.3528
	Patients	273	3.5	0.51			
EC	≤40 years	268	3.22	0.35	255	0.8708	0.1923
	>40 years	134	3.28	0.39			
	Physician and dietician	129	3.33	0.39	241	1.9321	0.0272*
	Patients	273	3.2	0.35			
PI	≤40 years	268	2.43	0.63	298	0.8268	0.2044
	>40 years	134	2.37	0.49			
	Physician and dietician	129	2.43	0.55	251	0.2905	0.3858
	Patients	273	2.41	0.61			

**Figure 4:**
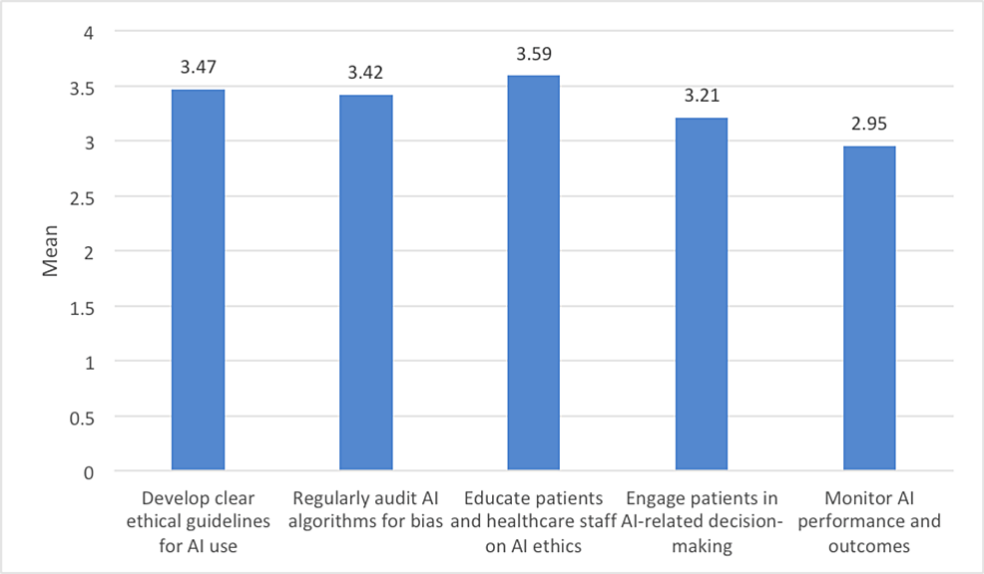
Image illustrating how artificial intelligence (AI) tools can enhance patient autonomy.

Figure 4 presents a set of recommendations for enhancing patient autonomy in the context of AI use in healthcare, each accompanied by an average rating. Notably, participants highly prioritized the development of clear ethical guidelines for AI use (3.47), indicating the significance of establishing a robust ethical framework to safeguard patient autonomy. Additionally, the recommendation to regularly audit AI algorithms for bias (3.42) received strong support, underlining the need to ensure fairness and equity in AI-driven healthcare decisions. Education played a key role, as participants emphasized the importance of educating both patients and healthcare staff on AI ethics (3.59) to enable informed decision-making and ethical AI implementation. However, the recommendation to engage patients in AI-related decision-making (3.21) received a slightly lower rating, suggesting room for improvement in involving patients actively. Monitoring AI performance and outcomes (2.95) was seen as important, though it received a lower rating, indicating that while monitoring is valued, it may not be as prioritized as other recommendations.

## Discussion

The purpose of this study was to explore the factors associated with AI and patient autonomy in obesity treatment decision-making. Given the novel nature of AI in healthcare, it is important to understand the physician-AI-patient relationships to better incorporate AI technologies in healthcare decision-making. Two out of the four factors from IPAS in this study demonstrated the widely recognized contrast between decision-making that prioritizes AI and decision-making that prioritizes the patient. One crucial element in the patient-physician-AI dynamic is the patient’s prerogative to determine whether to entrust the decision-making process to the AI system. The fourth principle pertains to the entitlement and responsibility of patients to be duly informed about pertinent hazards through the utilization of AI techniques in the field of healthcare.

Although diverse views were expressed by the participants in relation to the role of AI and patient autonomy in decision-making, it was emphasized that patients should be fully informed about the decisions, as well as the risks involved in the decisions provided by AI tools. These findings highlight the ethical and privacy concerns associated with the use of AI in healthcare treatment and decision-making in relation to various chronic conditions such as obesity, cancer, and mental health [25-31]. In addition, there are other factors such as legal issues emphasizing who takes responsibility if a treatment fails or has a negative effect which may result in serious issues. As there are no clear legal regulations [32], this may affect patient autonomy in decision-making as their right to know the full information may be compromised. Furthermore, the correlations between the IPAS scales indicate that no strong connections between patient autonomy and AI involvement, which may be due to the novel nature of AI in healthcare [33], the impact of which is not yet fully understood. Further supporting this the majority of the participants identified health literacy and access to information as the key influencing factors of patient autonomy. This can also be understood from the concerns as more than two-thirds of the participants identified the need for providing comprehensive information, ensuring access to health data and providing transparency as the major ways in which AI tools can enhance patient autonomy in obesity treatment and decision-making.

Accordingly, ethical and privacy concerns had a more negative impact on patient autonomy compared to trust and personal innovativeness. It has been observed that a lack of trust in AI is strongly associated with data privacy, transparency, explainability, usability, education, and patient safety [34-36], highlighting that trust is influenced by various factors in AI adoption. While age groups showed comparable levels of trust, physicians and dieticians expressed significantly higher trust in AI compared to patients (p < 0.05), indicating that healthcare professionals may have more confidence in AI tools for obesity treatment and decision-making. Accordingly, further supporting the trust influencing factors such as education, privacy, and transparency, the findings in this study suggested patient education and the development of ethical and regulatory guidelines as the key recommended approaches for enhancing patient autonomy in AI-enabled healthcare treatment and decision-making for obesity.

Implications

This study offers several theoretical and practical implications. The theoretical implications highlight the complex and nuanced relationship between AI and patient autonomy in healthcare decision-making, specifically in the context of obesity treatment. It underscores the need to balance the benefits of AI-driven personalized treatment strategies with the preservation of patient autonomy and ethical considerations. The study reveals varying perspectives on the role of AI, particularly in areas such as AI-assisted decision-making and patient information disclosure, and emphasizes the importance of transparency and patient involvement in the decision-making process. Furthermore, it highlights the role of trust, ethical concerns, and privacy risks in shaping patient autonomy and trust in AI. This study contributes to the growing body of literature on the intersection of AI and healthcare ethics, shedding light on the challenges and opportunities presented by AI in obesity treatment decision-making.

On a practical level, the findings offer valuable insights for healthcare providers, policymakers, and technology developers. The study suggests that clear ethical guidelines for AI use and regular audits of AI algorithms for bias are crucial for maintaining patient autonomy. Educating both patients and healthcare staff on AI ethics is vital for informed decision-making, and involving patients in AI-related decision-making should be encouraged. Additionally, monitoring AI performance and outcomes remains important, although it may require more emphasis. These practical recommendations can guide the development and implementation of AI technologies in healthcare, ensuring that they align with ethical principles, protect patient autonomy, and foster trust in AI among both healthcare providers and patients.

Limitations

A few limitations should be considered while interpreting the findings of this study. First, the study’s sample size, although substantial, may not fully represent the diversity of perspectives within the healthcare and patient populations. The majority of participants were from one region, potentially limiting the generalizability of the results to broader geographical areas and healthcare settings. Second, the study relied on self-reported data, which could introduce response bias or social desirability bias. Participants may have provided answers that they believed were more socially acceptable or aligned with ethical standards rather than reflecting their true beliefs and behaviors. Third, the study primarily focused on the perspectives of healthcare professionals and patients, potentially overlooking the insights of other stakeholders such as technology developers, policymakers, or ethicists who could provide additional perspectives on AI and patient autonomy. Lastly, the research primarily examined patient autonomy in the context of obesity treatment, and the findings may not be directly applicable to other healthcare domains. Future research should consider these limitations and work toward more comprehensive and diverse data collection to gain a more holistic understanding of AI’s impact on patient autonomy in various healthcare settings.

## Conclusions

In this empirical study, the complex interplay between AI and patient autonomy in the realm of obesity management was investigated. The study revealed a diverse range of perspectives on the extent to which AI should influence healthcare decision-making, underscoring the need to strike a delicate balance between the potential benefits of AI-driven personalized treatment strategies and the preservation of patient autonomy. Trust, ethical concerns, and privacy risks were identified as pivotal factors influencing patient autonomy in AI-enabled healthcare decision-making. The study’s practical implications emphasize the importance of establishing clear ethical guidelines, regular audits of AI algorithms, patient education, and patient involvement in AI-related decision-making to ensure that AI technologies in healthcare adhere to ethical principles, safeguard patient autonomy, and nurture trust. While the study has provided valuable insights, its limitations, including sample size and geographical representation, must be acknowledged. Nevertheless, this study lays the groundwork for further exploration of the evolving relationship between AI and patient autonomy, contributing to the ongoing discourse surrounding ethical considerations in AI-enhanced healthcare decision-making.
